# Superior Growth Strategies and Stable Rhizosphere Microbial Communities Enhance the Competitive Advantage of the Invasive Plant *Solanum rostratum* over Its Native Congener *S. nigrum*

**DOI:** 10.3390/plants15050687

**Published:** 2026-02-25

**Authors:** Yuanzhen Tang, Ping Guan, Meini Shao, Shuai Wang, Gue Liu, Ming Guan, Houyi Liu, Yuan Yang, Xiaolei Li, Jin Bai, Chenyang Xue, Bo Qu

**Affiliations:** 1 College of Bioscience and Biotechnology, Shenyang Agricultural University, Shenyang 110866, China; 2 Liaoning Provincial Agricultural and Rural Development Service Center, Shenyang 110001, China; 3 Liaoning Inspection, Examination & Certification Centre, Shenyang 110036, China; 4 Suizhong County Agricultural Affairs Service Center, Suizhong 125299, China; 5 College of Water Conservancy, Shenyang Agricultural University, Shenyang 110866, China

**Keywords:** interspecific competition, biological invasion, nitrogen deposition, microorganism

## Abstract

Exploring how nitrogen deposition alters the competitive interactions between invasive plants and native plants is critical for predicting the invasion trends of invasive plants and for formulating their control strategies. In this study, the invasive plant *Solanum rostratum* and its native congener *S. nigrum* were selected as research subjects, and three different nitrogen (N) concentration treatments (N1: 50 mg·kg^−1^, N2: 100 mg·kg^−1^, N3: 150 mg·kg^−1^) were set up to compare the two species in terms of growth and development, leaf nutrient utilization strategies, stress tolerance, and rhizosphere microbial community differences under competitive conditions. The results showed that the biomass of *S. rostratum* was 1.4 to 2.3 times that of *S. nigrum*; the former had a lower root–shoot ratio and a larger crown width, enabling it to seize more living space and light resources. Across all nitrogen treatments, the net photosynthetic rate of *S. rostratum* leaves was significantly higher than that of *S. nigrum*, reflecting a stronger carbon sequestration capacity. With the increase in soil nitrogen concentration, the malondialdehyde content in *S. rostratum* leaves showed a decreasing trend; meanwhile, its leaf soluble sugar and catalase contents were 3.5 to 4.3 times and 1.5 to 2.5 times those of *S. nigrum*, respectively, indicating a lower oxidative stress level and higher stress tolerance in *S. rostratum*. The leaf C/P and C/N ratios of *S. rostratum* increased with the rise in soil N, demonstrating a higher nutrient use efficiency, while the decrease in leaf phosphorus (P) content might be attributed to the element dilution effect caused by the rapid plant growth. In addition, the diversity and stability of the rhizosphere microbial community of *S. rostratum* gradually increased with increasing soil N and were significantly higher than those of *S. nigrum*. The rhizosphere-recruited microbes of the genera *Comamonas* and *Chryseobacterium* may help promote its root nutrient absorption and thus enhance its competitive ability. Collectively, our findings reveal that under exogenous N application, *S. rostratum* gains a significant growth advantage over *S. nigrum*, which is attributed to its stronger capacities for carbon assimilation and spatial resource acquisition, a nutrient strategy characterized by low acquisition and high utilization, as well as a stable and diverse rhizosphere microbial community.

## 1. Introduction

Biological invasion has inflicted severe impacts on the global economy and environment, emerging as a focal topic in environmental science and ecological research [1]. The global annual average direct and indirect economic costs caused by biological invasion amounted to 26.8 billion US dollars from 1970 to 2017, and this figure may have reached 46.8 billion US dollars in 2017 alone [2]. Literature statistics show that the number of invasive alien plant species in China increased from 58 to 515 between 1998 and 2018 [1]. It can be anticipated that China will continue to face high risks and harms from invasive alien plants for a considerable period of time.

Nitrogen (N) is one of the essential elements for plant growth and development; it alters plant community structure and ecosystem functions by modifying plant growth status and interspecific plant interactions. Reactive nitrogen input into terrestrial ecosystems via atmospheric deposition is recognized as one of the most pervasive drivers of global change [3]. Global total nitrogen deposition has shown a steady upward trend from 1977 to 2015, reaching as high as 92.7 teragrams of nitrogen (Tg N) across global terrestrial ecosystems by 2020 [4]. Against the backdrop of rising global nitrogen deposition, investigating the interactions between invasive plants and native plants not only deepens our understanding of the invasion mechanisms of invasive plants but is also critical to the future management and control of such invasive species [5].

N is an indispensable nutrient for plant growth and ecosystem productivity in both terrestrial and marine habitats. A wealth of studies has demonstrated that invasive plants exhibit positive responses to N deposition. These plants enhance their environmental adaptability and invasion potential by adjusting their growth strategies, altering interspecific interactions, modifying plant-insect interactions, and shaping soil microbial functions [6,7,8,9,10]. Under competitive conditions, high N treatment increases the biomass of the invasive plant *Duchesnea indica*; this species allocates more N to leaf tissues to boost shoot biomass, whereas its native congener *Fragaria vesca* prioritizes root biomass accumulation. Consequently, *D. indica* displays a strong competitive ability in N-rich environments [11]. The roots of the invasive plant *Alternanthera philoxeroides* are highly sensitive to N addition: On the one hand, it enhances nutrient uptake capacity by increasing root biomass and specific root length while reducing root diameter; on the other hand, it inhibits the growth and development of native plants by elevating allelochemical content [12]. At high nitrogen levels, the invasive grass *Melinis minutiflora* interferes with the native grass *Aristida riparia* in soil nitrogen acquisition, leading to a 52% decline in the latter’s relative growth rate [13]. For the perennial invasive vine *Mikania micrantha*, high N availability significantly enhances its carbon assimilation capacity, as well as its biomass and leaf physiological parameters, thereby strengthening its competitive ability in plant communities [14]. N deposition also markedly improves the insect herbivory resistance of *A. philoxeroides*, preserving its competitive advantage over the congeneric native species *A. sessilis* and potentially augmenting its invasion potential [15].

Furthermore, microbial communities play a pivotal role in mediating the invasion process of alien plants [16,17]. Native plant species develop species-specific microbial community characteristics through long-term evolution and environmental adaptation, and alien plant invasion may disrupt this ecological balance. During subsequent growth, plant–soil feedback loops tend to form positive effects for invasive species and negative effects for native species, ultimately leading to the gradual outcompetition of native species [18,19,20]. The invasive species *Brassica nigra* and *Bromus madritensis* show no significant differences in growth traits between sterilized and unsterilized soils, whereas the native species *Artemisia californica* and *Eriogonum fasciculatum* exhibit poor growth in sterilized soils. Under competitive conditions, invasive plants may enhance their competitive ability and invasion success by disrupting the stable microbial communities associated with native species [21]. Three dominant mycorrhizal fungal species (*Septoglomus viscosum*, *Septoglomus constrictum*, *Glomus perpusillum*) in the rhizosphere soil of the invasive plants *Ambrosia artemisiifolia* and *Bidens pilosa* can significantly promote plant N accumulation, strengthening their competitive advantage over the native grass *Setaria viridis* [22]. Invasive plants can also boost their competitive ability by recruiting beneficial microorganisms and modifying the rhizosphere nutrient environment [23,24]. The invasive cordgrass *Spartina alterniflora* accelerates soil N mineralization and dissimilatory nitrate reduction to ammonium (DNRA), increasing the supply of available ammonium N; meanwhile, its higher N uptake capacity allows it to outcompete salt marsh plants such as *Phragmites australis* [25]. Beyond root-associated microbes, foliar microbes also contribute substantially to the successful invasion of alien plants. N deposition reduces the pathogen infection rate of the invasive weed *A. philoxeroides*, and its phyllosphere endophytic microbes contain abundant antagonistic microbial taxa, which confers a distinct competitive advantage over the native *A. sessilis* under competitive conditions [26]. To date, few studies have explored the role of soil microbial communities in the competition between native and invasive plants under varying N levels. Such knowledge is nonetheless critical to predicting plant competitive interactions and the future trends of plant invasion worldwide.

Native to North America, *Solanum rostratum* has now spread to multiple provinces in China. It exhibits extremely strong reproductive capacity and environmental adaptability: A single plant can produce up to 10,000 seeds, enabling it to rapidly encroach on the living space of native plant species. In addition, *Solanum rostratum* degrades the forage quality of invaded grasslands. The entire plant is covered with sharp spines and contains solanine, a neurotoxin; accidental ingestion by livestock can lead to poisoning, indigestion, and even death, thereby posing a dual threat to the safety of agricultural and animal husbandry production as well as the ecological environment [27]. In this study, we selected the invasive plant *Solanum rostratum* and its native congener *S. nigrum* as research subjects, set three soil N levels, and recorded their growth and developmental traits under competitive conditions, with the aim of investigating the roles of plant growth strategies and soil microbes in enhancing the competitive advantage and invasion potential of *S. rostratum*. Specifically, we tested three hypotheses: (1) Under different nitrogen levels, *S. rostratum* exhibits superior growth and developmental traits compared with the native *S. nigrum*. (2) *S. rostratum* adopts more optimal growth and resource allocation strategies than its native congener *S. nigrum*. (3) With the increase in soil N concentration, the rhizosphere microbial communities of *S. rostratum* exhibit higher diversity and stability than those of *S. nigrum*.

## 2. Results

### 2.1. S. rostratum Outperforms Native S. nigrum in Biomass and Morphological Traits Under Competitive Conditions

**Figure 1:**
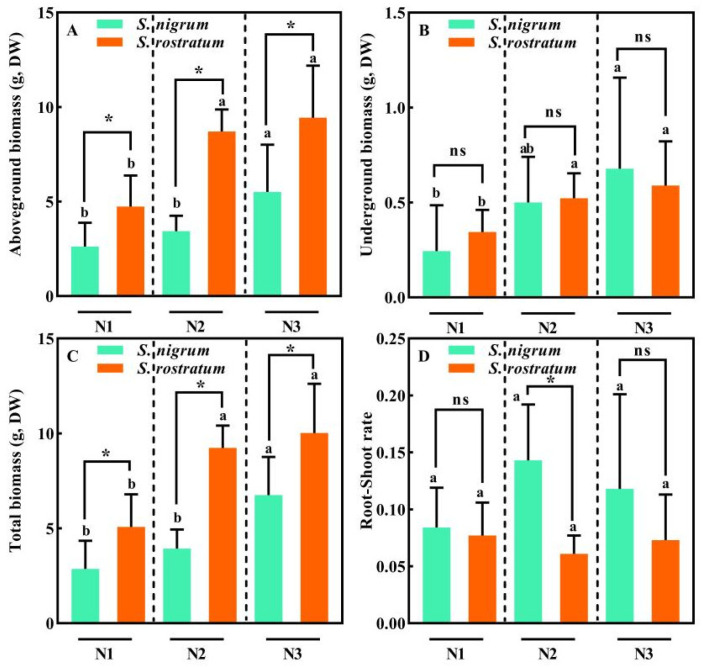
Biomass traits of *S. nigrum* and *S. rostratum* under different N levels. * indicates significant differences between *S. nigrum* and *S. rostratum* at the same N level (*p* < 0.05). (**A**) Aboveground biomass; (**B**) Underground biomass; (**C**) Total biomass; (**D**) Root-shoot rate. Different lowercase letters indicate significant differences among the three N levels for the same species (*p* < 0.05). ns—The difference was not statistically significant (ns). N1: Available N content 50 mg·kg^−1^; N2: Available N content 100 mg·kg^−1^; N3: Available N content 150 mg·kg^−1^. Replicates (n) = 9; error bars represent SD.

Soil N levels significantly affected plant growth and development, with the biomass of both *S. nigrum* and *S. rostratum* showing an increasing trend as soil N concentration rose (Figure 1). At the N1 and N2 levels, no significant differences were found in the biomass of all plant parts and the total biomass of *S. nigrum* (*p* > 0.05), while its biomass at the N3 level was significantly higher than that at the N1 and N2 levels (*p* < 0.05, Figure 1A–C). For *S. rostratum*, the biomass of all plant parts and the total biomass at the N1 level were significantly lower than those at the N2 and N3 levels (*p* < 0.05), and no significant difference in its biomass was observed between the N2 and N3 levels (*p* > 0.05, Figure 1A–C). Across all N treatments, the aboveground and total biomass of *S. rostratum* were significantly higher than those of *S. nigrum* (*p* < 0.05), whereas no significant difference in underground biomass was detected between the two species (*p* > 0.05). Soil N levels had no notable effect on the root–shoot ratio of either species, and no significant difference in root–shoot ratio was found between *S. nigrum* and *S. rostratum* across all N levels (*p* > 0.05, Figure 1D). Only at the N2 level did the root–shoot ratio of *S. nigrum* reach a significantly higher level than that of *S. rostratum* (*p* < 0.05). In comparison with *S. nigrum*, *S. rostratum* exhibited a lower root–shoot ratio and higher overall biomass, which indicated a better water balance achieved by *S. rostratum*.

**Figure 2:**
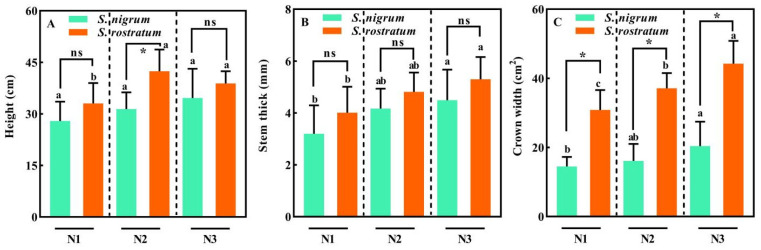
Morphological traits of *S. nigrum* and *S. rostratum* under different N levels. * indicates significant differences between *S. nigrum* and *S. rostratum* at the same N level (*p* < 0.05). (**A**) Height; (**B**) Stem thick; (**C**) Grown width. Different lowercase letters indicate significant differences among the three N levels for the same species (*p* < 0.05). ns—The difference was not statistically significant (ns). N1: Available N content 50 mg·kg^−1^; N2: Available N content 100 mg·kg^−1^; N3: Available N content 150 mg·kg^−1^. Replicates (n) = 9; error bars represent SD.

The plant height of *S. nigrum* at the N2 level was significantly lower than that of *S. rostratum* (*p* < 0.05), while no significant difference in plant height was observed between the two species at the N1 and N3 levels (*p* > 0.05, Figure 2A). Across all N levels, there was no significant difference in stem diameter between *S. rostratum* and *S. nigrum* (*p* > 0.05), and the stem diameter of both species increased markedly with the increase in nitrogen levels (Figure 2B). Interestingly, the canopy width of *S. rostratum* was significantly higher than that of *S. nigrum* under all N treatments (*p* < 0.05, Figure 2C). This implies that, in growth competition, the aboveground parts of *S. rostratum* can capture more living space and light resources by virtue of a larger canopy width, thereby enhancing its competitive ability.

### 2.2. S. rostratum Outperforms S. nigrum in Leaf Photosynthetic Capacity Under Competitive Conditions

**Figure 3:**
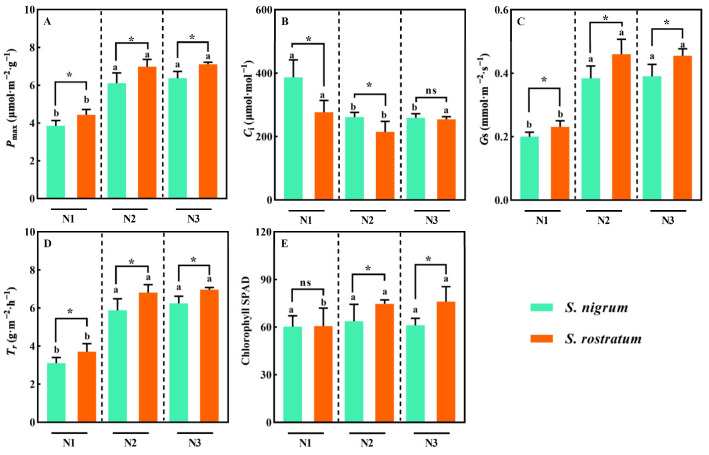
Photosynthetic traits of *S. nigrum* and *S. rostratum* under different N levels. * indicates significant differences between *S. nigrum* and *S. rostratum* at the same N level (*p* < 0.05). (**A**) The maximum net photosynthetic rate (*P*_max_); (**B**) The intercellular CO_2_ concentration (*C_i_*); (**C**) Stomatal conductance (*G_s_*); (**D**) Transpiration rate (*T_r_*); (**E**) The chlorophyll content. Different lowercase letters indicate significant differences among the three N levels for the same species (*p* < 0.05). ns—The difference was not statistically significant (ns). N1: Available N content 50 mg·kg^−1^; N2: Available N content 100 mg·kg^−1^; N3: Available N content 150 mg·kg^−1^. Replicates (n) = 9; error bars represent SD.

As soil N levels increased, the maximum net photosynthetic rate (*P*_max_), transpiration rate (*T_r_*), stomatal conductance (*G_s_*), and chlorophyll content of both species exhibited an increasing trend (Figure 3). At the N2 and N3 levels, the *P*_max_, *T_r_*, and *G_s_* of both species were significantly higher than those at the N1 level (*p* < 0.05, Figure 3A,C,D). However, the intercellular CO_2_ concentration (*C_i_*) of both species decreased with the rise in soil N levels (Figure 3B). Across all three N levels, the *P*_max_, *T_r_*, and *G_s_* of *S. rostratum* were significantly higher than those of *S. nigrum* (*p* < 0.05, Figure 3A,C,D). Meanwhile, at the N2 and N3 levels, the chlorophyll content of *S. rostratum* was significantly higher than that of *S. nigrum* (*p* < 0.05, Figure 3E). In competition with *S. nigrum*, *S. rostratum* displayed significantly higher values in multiple photosynthetic capacity indices, which reinforces the advantage of its aboveground canopy width and markedly elevates its overall competitive ability.

### 2.3. S. rostratum Outperforms S. nigrum in Leaf Tolerance Under Competitive Conditions

**Figure 4:**
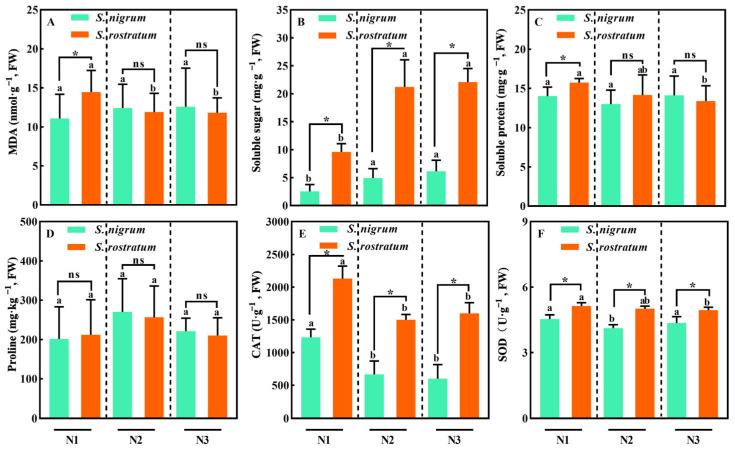
Leaf tolerance traits of *S. nigrum* and *S. rostratum* under different nitrogen levels. * indicates significant differences between *S. nigrum* and *S. rostratum* at the same N level (*p* < 0.05). (**A**) Malondialdehyde (MDA); (**B**) The soluble sugar content; (**C**) The soluble protein content; (**D**) proline content; (**E**) The catalase (CAT) activity; (**F**) The superoxide dismutase (SOD) activity. Different lowercase letters indicate significant differences among the three N levels for the same species (*p* < 0.05). ns—The difference was not statistically significant (ns). N1: Available N content 50 mg·kg^−1^; N2: Available N content 100 mg·kg^−1^; N3: Available N content 150 mg·kg^−1^. Replicates (n) = 9; error bars represent SD.

With the increase in soil nitrogen content, the malondialdehyde (MDA) content in *S. rostratum* leaves exhibited a decreasing trend, while that in *S. nigrum* leaves showed no significant change (Figure 4A). As soil N content increased, the soluble sugar content in the leaves of both species displayed a significant increasing trend, and the content in *S. rostratum* was significantly higher than that in *S. nigrum* (Figure 4B, *p* < 0.05). The soluble protein content, catalase (CAT) activity, and superoxide dismutase (SOD) activity in *S. rostratum* leaves all showed a decreasing trend, whereas only the CAT activity in *S. nigrum* leaves exhibited a decreasing trend. Additionally, the CAT and SOD activities in *S. rostratum* leaves were significantly higher than those in *S. nigrum* leaves (Figure 4C–F, *p* < 0.05). Overall, *S. rostratum* leaves exhibited a lower oxidative level and higher stress tolerance, which indicates that the environment was less stressful for *S. rostratum*.

### 2.4. S. rostratum Exhibits Higher Carbon Sequestration Capacity and Nutrient Use Efficiency Under Competitive Conditions

**Figure 5:**
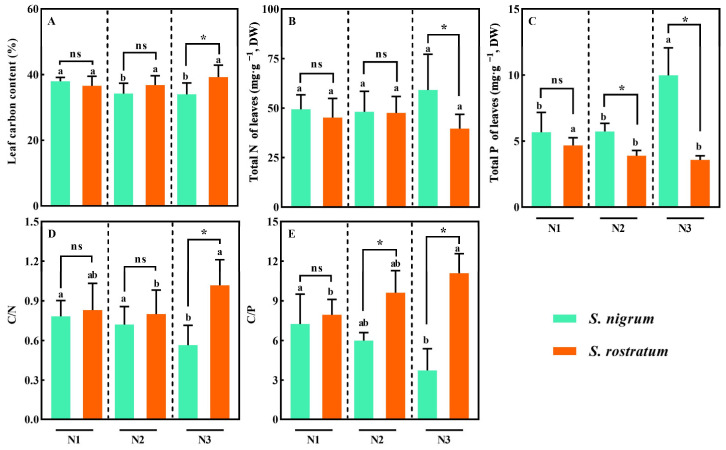
Leaf elemental contents of *S. nigrum* and *S. rostratum* under different nitrogen levels. * indicates significant differences between *S. nigrum* and *S. rostratum* at the same N level (*p* < 0.05). (**A**) Leaf carbon content; (**B**) Total N of leaves; (**C**) Total P of leaves; (**D**) C/N; (**E**) C/P. Different lowercase letters indicate significant differences among the three N levels for the same species (*p* < 0.05). ns—The difference was not statistically significant (ns). N1: Available N content 50 mg·kg^−1^; N2: Available N content 100 mg·kg^−1^; N3: Available N content 150 mg·kg^−1^. Replicates (n) = 9; error bars represent SD.

Significant differences in leaf carbon (C), nitrogen (N), and phosphorus (P) contents were observed between *S. rostratum* and *S. nigrum* across different N treatments. At the N3 level, the leaf N content of *S. nigrum* was significantly higher than that of *S. rostratum* (Figure 5A). Notably, the leaf P and C contents of *S. nigrum* and *S. rostratum* exhibited an opposite trend: with the increase in soil N content, the leaf P content of *S. nigrum* increased while its leaf C content decreased, whereas the leaf P content of *S. rostratum* decreased and its leaf C content increased (Figure 5B,C). At the N2 and N3 levels, the leaf P content of *S. nigrum* was significantly higher than that of *S. rostratum* (Figure 5B, *p* < 0.05). At the N3 level, the leaf C content of *S. rostratum* was significantly higher than that of *S. nigrum* (Figure 5C, *p* < 0.05). The leaf C/N and C/P ratios of *S. rostratum* and *S. nigrum* followed a consistent pattern: the ratios increased in *S. rostratum* but decreased in *S. nigrum* across increasing N levels (Figure 5D, F, *p* < 0.05). In competitive environments, *S. nigrum* and *S. rostratum* adopted diverging growth and development strategies, with the latter exhibiting higher carbon sequestration capacity and nutrient use efficiency.

### 2.5. S. rostratum Outperforms S. nigrum in Rhizosphere Soil Microbial Community Diversity and Stability Under Competitive Conditions

**Figure 6:**
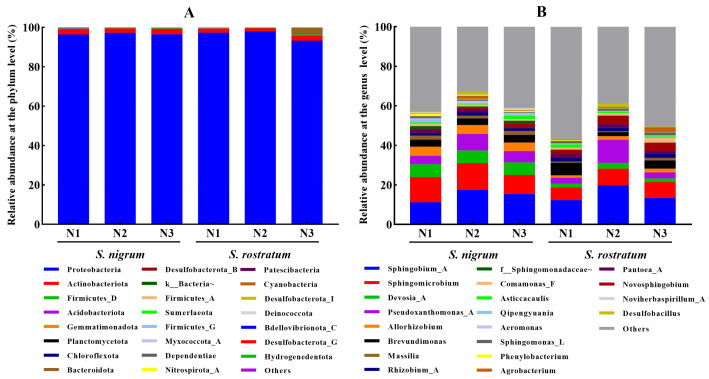
Variations in rhizosphere soil microbial communities of *S. nigrum* and *S. rostratum* under different N levels. (**A**) Relative abundance of microorganisms at the phylum level under different N concentrations; (**B**) Relative abundance of microorganisms at the genus level under different N concentrations.

We collected rhizosphere soil samples of *S. rostratum* and *S. nigrum* from the competition experiment to determine the soil microbial community structure and diversity. The results showed that at the phylum level, the relative abundance of only *Proteobacteria* in the rhizosphere soil of both species exceeded 90% (Figure 6A). With the increase in soil N levels, the relative abundance of *Proteobacteria* in the rhizosphere of *S. rostratum* decreased from 97.34% to 93.28%, while that of *Acidobacteriota* and *Bacteroidota* increased to varying degrees, with *Bacteroidota* showing the most remarkable increase, rising from 0.18% to 3.07%. However, across different N levels, the abundances of the top three dominant phyla (*Proteobacteria*, *Actinobacteriota*, and *Firmicutes_D*) in the rhizosphere of *S. nigrum* showed no significant changes (Figure 6A). At the genus level, with the increase in soil N levels, the relative abundances of such microbial genera as *Sphingomicrobium*, *Allorhizobium*, *Massilia*, *Novosphingobium*, and *Comamonas_F* in the rhizosphere of *S. rostratum* increased, whereas those of *Brevundimonas*, *Pantoea_A*, and *Asticcacaulis* exhibited a decreasing trend (Figure 6B). In contrast, with the increase in soil N levels, the relative abundances of most microbial genera in the rhizosphere of *S. nigrum* showed a decreasing trend, with only *Sphingobium_A*, *Pseudoxanthomonas_A*, and *Rhizobium_A* exhibiting an increasing trend (Figure 6B).

**Figure 7:**
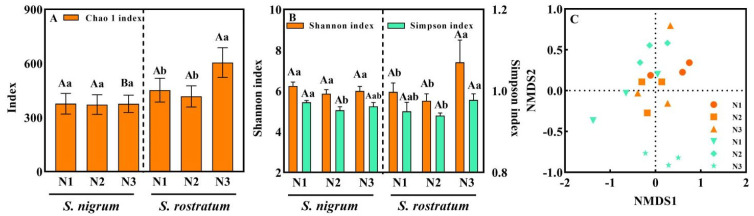
Microbial diversity, abundance indices and NMDS analysis of rhizosphere soil of *S. nigrum* and *S. rostratum* under different N levels. (**A**) Chao 1 index; (**B**) α diversity index; (**C**) NMDS analysis. Different lowercase letters indicate significant differences among different N concentrations for the same species (*p* < 0.05), and different uppercase letters indicate significant differences between *S. nigrum* and *S. rostratum* at the same N concentration (*p* < 0.05). Replicates (n) = 9; error bars represent SD.

With increasing N levels, the Chao 1 index of rhizosphere soil microorganisms of *S. nigrum* showed no significant change (Figure 7A), whereas that of *S. rostratum* exhibited an increasing trend and was significantly higher than that of *S. nigrum* at the N3 level (Figure 7A). Across different N levels, the Shannon index of rhizosphere microorganisms of *S. nigrum* had no significant variation, while its Simpson index showed a decreasing trend (Figure 7B). In contrast, both the Shannon and Simpson indices of rhizosphere microorganisms of *S. rostratum* increased significantly with the rise in N levels (Figure 7B). At the N3 level, the Shannon index of rhizosphere microorganisms of *S. rostratum* was higher than that of *S. nigrum* (Figure 7B). Results of NMDS analysis revealed that *S. nigrum* and *S. rostratum* showed a relatively close distribution, with an obvious separation only at the N3 level, which was consistent with the α-diversity indices (Figure 7C). In the same growth environment, the rhizosphere soil microorganisms of *S. nigrum* and *S. rostratum* had certain similarities in community structure, species diversity and spatial distribution; however, the higher Chao 1 index and Shannon index of the rhizosphere microbial community in *S. rostratum* may confer it a stronger competitive advantage.

**Figure 8:**
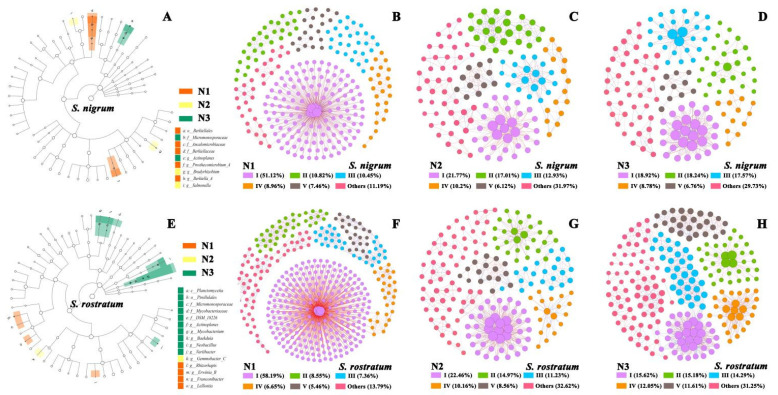
LDA Effect Size (LEfSe, LDA ≥ 2, *p* value < 0.05) and co-occurrence networks of rhizosphere soil microbial communities of *S. nigrum* and *S. rostratum* under different N levels at the OTU level (│Pearson’s correlation│ ≥ 0.9, and *p* value < 0.05). (**A**,**E**) LEfSe analysis of rhizosphere soil microbial communities of *S. nigrum* and *S. rostratum*; (**B**–**D**) Co-occurrence networks of rhizosphere soil microorganisms of *S. nigrum* under different N concentrations; (**F**–**H**) Co-occurrence networks of rhizosphere soil microorganisms of *S. rostratum* under different N concentrations.

At the phylum level, neither *S. nigrum* nor *S. rostratum* exhibited significantly enriched microbial taxa (Figure 8A,E). With increasing N levels, the number of significantly enriched microbial taxa in the rhizosphere soil of *S. nigrum* decreased. At the N1 level, *Ancalomicrobiaceae* and *Berkiellaceae* at the family level, as well as *Prosthecomicrobium-A* and *Berkiella-A* at the genus level, were significantly enriched; at the N3 level, only *Micromonosporaceae* at the family level and *Actinoplanes* at the genus level were significantly enriched (Figure 8A). In contrast, the rhizosphere microorganisms of *S. rostratum* showed the opposite pattern, with the number of significantly enriched microbial taxa at the N3 level being significantly higher than that at the N1 level (Figure 8E). At the N1 level, *Rhizorhapis*, *Erwinia-B*, *Franconibacter* and *Lelliottia* at the genus level were enriched; at the N3 level, *Micromonosporaceae*, *Mycobacteriaceae* and *DSM-18226* at the family level, as well as *Actinoplanes*, *Mycobacterium*, *Baekduia*, *Neobacillus* and *Variibacter* at the genus level, were significantly enriched (Figure 8E). LEfSe analysis revealed that the rhizosphere microbial communities of *S. nigrum* and *S. rostratum* exhibited distinct response characteristics to N addition: *S. nigrum* enriched more differential microbial taxa under low N levels, while *S. rostratum* did so under high N levels.

For all N levels, the rhizosphere microbial co-occurrence network analyses of *S. nigrum* and *S. rostratum* showed that the modularity indices of all network plots were greater than 0.45, indicating a significant modular structure of the networks (Supplementary Table S1, Figure 8B–D,F–H). With increasing N levels, the number of nodes (421–224), network diameter (33–22), average path length (10.51–8.21) and number of edges (2075–1261) in the rhizosphere soil microbial co-occurrence network of *S. rostratum* all decreased gradually, while the average degree (19.72–22.52) increased (Figure 8F–H, Supplementary Table S1); among these metrics, the proportion of positive correlations decreased from 81.73% to 59.87%. For *S. nigrum*, with the rise in N levels, the number of nodes (268–148), network diameter (18–12), average degree (19–12.81) and number of edges (1273–474) in its rhizosphere soil microbial co-occurrence network all decreased gradually (Figure 8B–D, Supplementary Table S1). These results indicated that in the competitive environment between *S. rostratum* and *S. nigrum*, the interactions among rhizosphere soil microorganisms were strengthened and microbial stability was enhanced with the increase in soil N levels. In addition, the modularity, average degree, number of nodes, number of edges and proportion of positive correlations of the rhizosphere microbial co-occurrence network of *S. rostratum* were significantly higher than those of *S. nigrum*. This demonstrated that the rhizosphere microbial community interactions of *S. rostratum* were overall superior to those of *S. nigrum* in the competitive relationship, which may enhance the competitive ability of *S. rostratum* by improving nutrient cycling in the plant rhizosphere and nutrient uptake by the root system (Figure 8).

**Figure 9:**
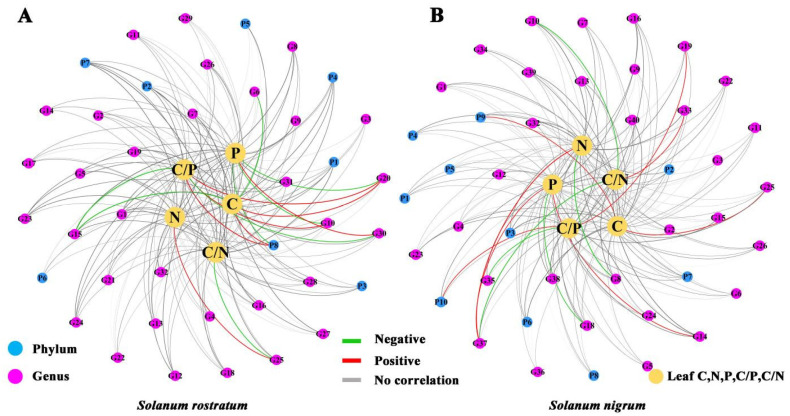
Relationships between rhizosphere soil microbial communities and leaf nutrient contents of *S. nigrum* and *S. rostratum* under different N levels. Detailed information on the phyla and genera is provided in Supplementary Table S2. (**A**) *S. nigrum*; (**B**) *S. rostratum*.

There was no significant difference in the number of interaction relationships between rhizosphere microorganisms and leaf nutrient contents of *S. rostratum* and *S. nigrum*. *S. rostratum* had seven positive correlations and eight negative correlations, while *S. nigrum* had eight and five, respectively. However, the microbial taxa involved in the correlation networks of the two species were significantly different. In *S. rostratum*, both leaf C content and C/P ratio were significantly positively correlated with P8 (P-Others); leaf C/P ratio and P content exhibited significant interactions with G10 (*Comamonas_F*), G15 (*Achromobacter*), G20 (*Chryseobacterium*) and G30 (*Rhizorhapis*); and leaf C/N ratio and N content had significant interactions with G25 (*Acidovorax_A*) (Figure 9A). In *S. nigrum*, leaf C content and C/P ratio were significantly positively correlated with P9 (*Patescibacteria*) and P10 (*Bdellovibrionota_E*), respectively; leaf C, N, P contents, as well as C/N and C/P ratios, showed significant interactions with G14 (*Asticcacaulis*), G19 (*Acinetobacter*), G25 (*Acidovorax_A*), G33 (*Cupriavidus*) and G37 (*Pseudoduganella*) (Figure 9B). These results indicated that each species harbors a species-specific rhizosphere microbial community, which may correspond to their distinct growth strategies.

## 3. Discussion

### 3.1. S. rostratum Outperforms S. nigrum in Growth and Development Capacity as Well as Stress Tolerance Under Competitive Conditions

Interactions between invasive and native plants play a pivotal role in regulating community dynamics; they not only determine the composition of plant communities but also gradually alter resource availability and habitat structure in ecosystems [28]. Such interactions are influenced by external environmental factors, and exploring how they respond to environmental drivers, as well as the potential ecological impacts of these responses, is of great practical significance for elucidating the invasion mechanisms of invasive plants and developing targeted control measures [29].

Numerous studies have confirmed that exogenous N addition promotes the growth and development of both alien invasive plants and native plants. When growing, invasive plants tend to exhibit more positive responses, whereas the growth of native plants is often inhibited. In competition, the aboveground parts of invasive plants typically enhance photosynthetic capacity and increase biomass allocation to compete for growth space and light resources, maintaining a higher growth rate and further strengthening their growth advantage. In contrast, their underground parts adopt an opposite strategy: with the reduced difficulty of N acquisition in soil, invasive plants decrease root biomass allocation to lower energy consumption while increasing biomass allocation to assimilatory organs [30]. For instance, under competitive conditions, exogenous N application promoted the growth of invasive plants (*Erigeron annuus*, *Conyza canadensis*, *Amaranthus retroflexus*) and native plants (*Artemisia argyi*, *Inula japonica*, *Achyranthes bidentata*), while the invasive plants tended to allocate more biomass to aboveground parts to improve their competitive ability by competing for light resources [31]. The net photosynthetic rate of *Ageratina adenophora* is higher than that of its native related species *Eupatorium heterophyllum* and *E. japonicum*; this high net photosynthetic rate endows *A. adenophora* with a faster growth rate and greater total biomass. Its strategy of lower root biomass allocation reduces energy consumption, and its larger plant size enables it to seize more space and light resources [32]. In addition, the competitive advantage of invasive and native plants is also influenced by N forms: Invasive plants (*Xanthium strumarium*, *Ambrosia trifida*, *Bidens frondosa*) exhibit a more pronounced growth advantage over their native related species, and this advantage is more significant under nitrate N conditions [33].

Notably, the results of this study showed that plant antioxidant enzyme activities decreased with the increase in soil N levels; both SOD and CAT activities in *S. rostratum* and *S. nigrum* exhibited a decreasing trend, with the former being significantly higher than the latter. Relevant studies have indicated that exogenous ammonium nitrate application enhanced the competitive advantage of *Aegilops tauschii*, whose SOD activity and MDA content both increased with rising N concentrations [34]. Under competitive conditions, with the increase in soil N concentrations, leaf antioxidant enzyme activities of the invasive species *Wedelia trilobata* and the native species *W. chinensis* showed an increasing trend, and the POD activity of the former was significantly higher than that of the latter [35]. Numerous studies on plant stress caused by abiotic and biotic factors have demonstrated that antioxidant enzyme activities tend to increase with the intensification of stress when the stress level does not exceed the plant’s tolerance threshold [29,36]. N is not a toxic substance for plants, and the continuous increase in plant biomass in this study indicated that the plants did not suffer from high N stress. The soil moisture content in this experiment was set to 35–45% of field capacity (a moderate drought level), which may have subjected the plants to a stressed state, while the increase in soil N levels alleviated such stress. Furthermore, consideration should be given to the interactive effects of water and nutrients on plant traits, as plant traits may exhibit significant differences in their responses under different water gradients. Compared with *S. nigrum*, *S. rostratum* exhibited a more positive response to N addition: its oxidative level, soluble protein content, and antioxidant enzyme activities all showed a decreasing trend, yet the continuously increasing soluble sugar content still maintained its high stress tolerance. N fertilizer may alleviate the effects of drought on plants by maintaining normal plant physiological characteristics and scavenging reactive oxygen species (ROS) generated by drought stress [37,38,39]. For example, under drought stress, when the N application rate increased from 90 kg N·hm^−2^ to 120 kg N·hm^−2^, the leaf antioxidant enzyme activities of three *Sorghum bicolor* cultivars decreased significantly [40]. Under mild soil water stress (55–60% moisture content), exogenous urea application effectively reduced the production of free radicals in sorghum and maize, decreased antioxidant enzyme activities, and alleviated the stress effects of water deficit on plants [41]. Similar findings have also been reported in studies on *Triticum aestivum* [42], *Pinus massoniana* [43], and *Leymus chinensis* [44].

In this study, under different N concentration treatments, the total biomass, *P_max_*, leaf chlorophyll content, and crown width of *S. rostratum* were significantly higher than those of the native species *S. nigrum*. This indicated that, like other invasive plants, *S. rostratum* exhibited a higher carbon assimilation capacity and spatial resource acquisition ability through its aboveground growth strategy, which ensured its growth advantage under competitive conditions. Meanwhile, the higher antioxidant enzyme activities and soluble sugar content enhanced the ability of *S. rostratum* to resist environmental changes.

### 3.2. S. rostratum Possesses a Superior Nutrient Utilization Strategy Under Competitive Conditions

Compared with native species, invasive species may exhibit higher carbon assimilation capacity, resource acquisition and utilization efficiency, and other superior traits, which are reflected in their leaf traits as enhanced photosynthetic capacity, elevated carbon assimilation rate, increased leaf nitrogen content, and related attributes. These traits enable them to break free from the trade-off in functional traits that restricts coexisting native species [45]. In this study, with the increase in soil N concentration, the leaf N content of *S. rostratum* showed no significant change, while its leaf P content exhibited a decreasing trend; additionally, under the N3 treatment, the leaf N concentration of *S. nigrum* was significantly higher than that of *S. rostratum*. This phenomenon may be attributed to the dilution effect of elemental concentrations: when the rate of plant dry matter accumulation exceeds that of elemental accumulation, nutrient concentrations will decrease [46]. In competitive interactions between the invasive plant *Oenothera biennis* and three native species (*Artemisia argyi*, *Inula japonica*, *Chenopodium album*), leaf N concentration of *O. biennis* showed no significant change with increasing soil N concentration, whereas its leaf P concentration decreased significantly [47]. A study investigating the growth of 30 native and invasive annual plant species under high and low N conditions revealed that invasive plants had significantly lower leaf N content but higher C/N ratios under high N conditions; the researchers proposed that fast-growing species may improve overall productivity by diluting leaf nitrogen [48]. In addition, relevant studies have confirmed that exogenous N addition under competitive conditions increases the leaf P concentration of the invasive plant *Solidago canadensis* and the native plant *Pterocypsela laciniata*. This may be because exogenous N application increases soil acidity, promotes the dissolution of soil P, and thus enhances plant P uptake [49]. However, this mechanism cannot well explain the increase in leaf P content of *S. nigrum* observed in this study. The Leaf Economic Spectrum (LES) classifies plants into two functional types: the fast investment-return type and the slow investment-return type. The former typically has stronger photosynthetic capacity and higher nutrient utilization efficiency, which reflects the trade-off between leaf construction cost and plant growth [50]. Leaf chemical traits are one of the key indicators reflecting plant growth strategies [51]. Global surveys of invasive plant leaf traits have shown that invasive species possess more efficient nutrient utilization strategies than native species [16]. In this study, with the increase in N concentration, both the leaf C/N and C/P ratios of *S. rostratum* increased significantly, while those of *S. nigrum* showed the opposite trend. This indicates that *S. rostratum* has higher carbon sequestration capacity and nutrient utilization efficiency, enabling it to fully utilize environmental resources and embodying a “low acquisition-high utilization” ecological strategy [52]. Furthermore, *S. rostratum* has dense stiff spines on its leaves and stems, and its relatively high C/N ratio may be associated with the synthesis of structural substances required for spine formation [53,54].

### 3.3. S. rostratum Has Superior Rhizosphere Microbial Community Diversity and Stability Under Competitive Conditions

Soil biota play a pivotal role in shaping plant communities and maintaining terrestrial ecosystem functions [55,56]. Numerous studies have demonstrated that soil microorganisms alter the probability of successful invasion of invasive plants by participating in multiple ecological processes, such as plant nutrient cycling and absorption, pest and disease control, and insect herbivory [57,58,59,60]. The interaction between invasive plants and soil factors jointly promotes the growth performance of invasive plants through positive feedback. Under competitive conditions, exogenous N addition exerts positive soil feedback on the invasive tree species *Rhus typhina*, significantly increasing the number of its root-associated microorganisms, while the native species *Ailanthus altissima* shows the opposite result. Therefore, nitrogen deposition and interspecific competition may enhance the competitive advantage of invasive plants through plant–soil feedback [61]. Invasion by *A. adenophora* significantly increases the abundance of soil arbuscular mycorrhizal fungi (AMF), and soil feedback experiments have shown that invaded soil has a significant inhibitory effect on the growth of native plants [62]. In addition, *Bacillus* sp. ScRB44 isolated from the rhizosphere of the invasive plant *Solidago canadensis* has a strong ability to produce indole-3-acetic acid (IAA), can significantly improve nitrogen use efficiency, and has a significant promoting effect on the growth of *S. canadensis* under nutrient-deficient conditions [63]. In competitive conditions, the abundance of *Bacillus idriensis* in the rhizosphere soil of *A. adenophora* shows the greatest increase; inoculation with *B. idriensis* increases the biomass of *A. adenophora* by 175.83% under intercropping conditions, thereby enhancing its competitive advantage over the native plant *Rabdosia amethystoides* [58]. In this study, the results of the competition experiment showed that exogenous N addition increased the α-diversity index of the microbial community in the rhizosphere soil of *S. rostratum*. Meanwhile, exogenous N application enhanced the stability of rhizosphere soil microorganisms between *S. rostratum* and *S. nigrum*. Furthermore, the modularity, average degree, number of nodes, number of edges, and proportion of positive correlations of the rhizosphere microbial co-occurrence network of *S. rostratum* were significantly higher than those of *S. nigrum*. This indicates that in the competitive relationship, the rhizosphere microbial community interactions of *S. rostratum* are overall superior to those of *S. nigrum*, which may enhance the competitive ability of *S. rostratum*.

Phosphorus (P) is one of the essential macronutrients for plant growth and development, yet the low bioavailability of soil P limits plant uptake. Therefore, plants often recruit specific beneficial microorganisms to enhance their absorption of soil nutrients and promote their own growth [24,64]. For instance, studies have shown that *Achromobacter* sp. FB-14 exhibits significant 1-aminocyclopropane-1-carboxylate (ACC) deaminase activity, indole compound synthesis capacity, and phosphate-solubilizing ability. After inoculation in rice, it alleviates salt stress in rice by upregulating the expression of stress-responsive CIPK genes (OsCIPK03, OsCIPK12 and OsCIPK15) [65]. *Achromobacter* strains isolated from eutrophic aquatic ecosystems possess strong phosphate-solubilizing functions; OPMB3, a lecithin-mineralizing bacterium, exhibits robust phosphate-mineralizing ability under laboratory culture conditions [66]. In this study, the leaf P content of *S. rostratum* decreased significantly. We speculate that one of the reasons may be the “dilution effect”; in addition, rhizosphere microorganisms may also contribute to the reduction in P content, as both the leaf C/P ratio and C content showed a significant negative correlation with G15 (*Achromobacter*). Furthermore, beneficial microorganisms *Pseudoduganella* and *Chryseobacterium* recruited in the plant rhizosphere can promote plant growth and development [67]. For example, maize with abundant *Pseudoduganella* enrichment in the rhizosphere has higher aboveground and root biomass [68]. *Chryseobacterium* strains can promote the growth of sunflower and chrysanthemum and alleviate water stress experienced by these plants [69,70,71]. In this study, both the leaf C/P ratio and C content of *S. rostratum* showed a significant positive correlation with G20 (*Chryseobacterium*); the abundance of G37 (*Pseudoduganella*) in the rhizosphere of *S. nigrum* exhibited a significant positive correlation with leaf N and P contents. However, based on the results presented in this study, we cannot directly confirm a direct causal relationship between plant element contents and specific microorganisms. According to the existing correlation results and relevant literature, it can only indicate an association between the two. Future studies should supplement more data on plant nutrient uptake capacity and microbial functions.

Although the results of our experiment indicated that *S. rostratum* gained an advantage in the competition with *S. nigrum*, more systematic investigations should be conducted in future studies. First, a single nitrogen source (urea) was used in this study, while plants exhibit obvious preferential characteristics for N forms. For example, in the competition experiment between the invasive plant *B. alba* and its native relative *B. biternata*, NH_4_^+^ would weaken the competitive advantage of *B. alba*, whereas NO_3_^−^ would enhance it [72]; the invasive plant *X. strumarium* prefers to take up nitrate N, while its native relative *X. sibiricum* prefers to take up ammonium N [73]. The experimental results may change when different N forms are adopted. Second, the environmental factors and pot conditions were set as single variables. In natural field conditions, plants are often exposed to multi-factor stresses such as herbivory, nutrient and water stress, and soil pathogen infection. This study only considered a single nitrogen source and lacked monoculture pots, which cannot comprehensively reflect the real results during plant competition or accurately calculate plant competition indices. Whether the results of this pot experiment are consistent with field realities still requires careful evaluation. Finally, the research on plant rhizosphere microorganisms in this study was limited to bacterial communities and lacked more detailed verification experiments; it did not consider the roles of fungi, mycorrhizal fungi, and nematodes in root nutrient absorption and competition [74,75], which is not conducive to identifying the key factors governing the competitive outcome. In general, to verify and determine the competitive relationship between invasive plants and native plants, it is necessary to base the research on natural ecological environments and improved experimental designs.

## 4. Materials and Methods

### 4.1. Experimental Materials and Overview of the Experimental Site

The experimental materials used in this study were the invasive plant *S. rostratum* and the congeneric native plant *S. nigrum*, which were collected from Kailu County, Tongliao City, Inner Mongolia Autonomous Region in 2023. The collected seeds were stored in a cool and well-ventilated place for subsequent use. The experimental site was located at the Scientific Research Base of Shenyang Agricultural University (41°50′ N, 123°34′ E). In April 2024, seed germination and seedling cultivation of *S. rostratum* and *S. nigrum* were carried out in a greenhouse. In May 2024, seedlings with uniform plant size and a height of approximately 10 cm were selected as research materials, and the two species were planted together in one pot.

### 4.2. Experimental Design

Based on field investigations, relevant literature and the previous research results of our research group, the soil moisture content in this study was set to 35–45% of the field capacity (at a moderate drought level). Three soil N addition levels were established, namely N1 (50 mg·kg^−1^), N2 (100 mg·kg^−1^) and N3 (150 mg·kg^−1^), with 9 replicates for each treatment. Soil field capacity was determined in the laboratory using the core cutter method. The pots were weighed every evening, and the corresponding amount of water was supplemented based on the measured results. Soil physicochemical properties: pH 7.6, total nitrogen 0.47 g·kg^−1^, available nitrogen 10.24 mg·kg^−1^, organic matter 1.23 g·kg^−1^, and total phosphorus 0.26 g·kg^−1^. The plastic pots used in the experiment were 17 cm × 23 cm in size, and each pot was filled with 3.5 kg of soil. In April 2024, the soil N levels were regulated by urea application, which was conducted prior to seedling transplantation (applied in three split doses at 7-day intervals, thoroughly mixed with the soil, and then the soil was watered to 35–45% of the field capacity).

### 4.3. Determination of Plant Morphological and Growth Indices

Plant height was measured with a ruler (cm), and stem diameter was determined with a vernier caliper (mm). The dry weights of the aboveground and underground parts were measured by oven-drying the samples at 80 °C followed by weighing. Based on these measurements, total biomass (aboveground parts + underground parts) and root-to-shoot ratio (R/S, root biomass/aboveground biomass) were calculated. The leaves used for chlorophyll content determination were also included in the statistics of leaf area, leaf biomass and total biomass.

### 4.4. Determination of Plant Photosynthetic Characteristic Indices

The maximum net photosynthetic rate (*P*_max_) of plant leaves was measured using a Li-6800 Portable Photosynthesis System (Li-Cor, Inc., Lincoln, NE, USA), and the intercellular CO_2_ concentration (*C_i_*), transpiration rate (*T_r_*), and stomatal conductance (*G_s_*) were obtained from the instrument output. A red-blue light source was adopted for the measurements, with the photosynthetic photon flux density in the leaf chamber set to 1500 μmol·m^−2^·s^−1^, the CO_2_ concentration set to 380 μmol·mol^−1^, and the leaf temperature and relative humidity maintained at 28 °C and 55%, respectively. Healthy third or fourth leaves from the top to the bottom of the plants were selected for the measurements. The chlorophyll content was determined with a SPAD-502Plus Chlorophyll Meter (Konica Minolta, Inc., Tokyo, Japan), and efforts were made to select the same leaves used for the photosynthetic characteristic measurements to ensure experimental consistency.

### 4.5. Determination of Nutrient Contents in Plant Leaves

The carbon and nitrogen contents in plant leaves were determined via the potassium dichromate oxidation method (T/NAIA 070-2021). Fresh leaves were sampled, de-enzymed in an oven at 105 °C for 30 min, and then dried at 55 °C to constant weight. An accurately weighed 20.00 mg of the dried sample was placed into a 25 mL hard glass test tube, to which 10 mL of 0.4 mol/L potassium dichromate-sulfuric acid solution was added. The test tube was sealed with a cover and soaked for 48 h and then heated in a digestion furnace at 245 °C for 5 min of boiling. After the sample solution cooled to room temperature, it was quantitatively transferred into a 150 mL Erlenmeyer flask, with 2 drops of o-phenanthroline indicator added subsequently. The solution was titrated with a 0.2 mol/L standard ferrous sulfate solution, and the endpoint of titration was defined as the color change of the solution from orange-yellow to blue-green and finally to reddish-brown. If the volume of ferrous sulfate solution consumed for sample titration was less than one-third of that consumed for blank titration, the sample weight should be reduced to ensure the complete oxidation of carbon. For blank experiments, 20.00 mg of silica was used to replace the sample.

(1) $$X=\frac{(V_{0}-V)\times C\times 0.003}{m}\times 100$$

where X = total carbon content on a dry weight basis (%); V_0_ and V = volumes of standard ferrous sulfate solution consumed for blank and sample titration, respectively (mL); C = concentration of ferrous sulfate solution (mol/L); m = mass of the sample (g); 0.003 = millimolar mass of one-fourth of a carbon atom (g).

The nitrogen content in plant leaves was determined using an Organic Elemental Analyzer vario MACRO cube (Elementar Analysensysteme GmbH, Langenselbold, Germany), with reference to Determination of Nitrogen, Phosphorus and Potassium in Plants (NY/T 2017-2011). The phosphorus content in plant leaves was determined via the molybdenum-antimony anti-spectrophotometric method (NY/T 2017-2011). Fresh leaves were de-enzymed in an oven at 105 °C for 30 min and then dried at 55 °C to constant weight. A total of 0.2 g of the dried sample was weighed into a crucible, to which 5 mL of sulfuric acid and 4 mL of hydrogen peroxide were added. After the violent reaction ceased, the crucible was placed on a digestion furnace and heated for digestion until the solid matter dissolved completely to form a solution. Heating was stopped when white fumes of sulfuric acid appeared and the solution turned brown. After cooling slightly, 10 drops of hydrogen peroxide were added, and heating for digestion was continued for approximately 5 min. The crucible was then cooled, another 10 drops of hydrogen peroxide were added for further digestion, and this process was repeated until the solution became colorless or clear, followed by an additional 5 min of heating. After cooling to room temperature, the solution was made up to a constant volume of 100 mL with distilled water, filtered through filter paper, and stored for subsequent determination. A series of standard phosphorus solutions were prepared, the absorbances of which were measured at a wavelength of 700 nm using a spectrophotometer, and the linear regression equation was calculated for phosphorus content quantification.

(2) $$\omega =\frac{\rho \times V}{m}\times \frac{V_{2}}{V_{1}}\times 10^{-5}$$

where ρ = total phosphorus concentration of the test solution (mg/L); V = volume of the test solution made up to a constant volume (mL); V_1_ = volume of the test solution pipetted for analysis (mL); V_2_ = volume of the color-developed solution made up to a constant volume (mL); m = mass of the sample (g).

### 4.6. Determination of Plant Osmotic Adjustment Substances and Antioxidant Enzyme Activities

The malondialdehyde (MDA) content in plant leaves was determined via the thiobarbituric acid method; the soluble sugar content was determined by the phenol-sulfuric acid colorimetric method; the soluble protein content was measured using the Coomassie brilliant blue staining method; the free proline content was assayed by the ninhydrin colorimetric method; the catalase (CAT) activity was determined through the ultraviolet absorption method; the superoxide dismutase (SOD) activity was measured via the nitroblue tetrazolium (NBT) photochemical reduction method [76,77].

### 4.7. Determination of Soil Microbial 16S rRNA

Root systems were separated from soil using destructive sampling, with manual separation of soil and roots. Soil samples for microbial community analysis were collected from soil within 2 mm of the plant roots. The samples were stored in liquid nitrogen containers, then preserved in an ultra-low-temperature freezer at −80 °C, and finally shipped to the sequencing company in dry ice boxes. Total genomic DNA samples were extracted using the MagBeads FastDNA Kit for Soil (116564384; MP Biomedicals, Santa Ana, CA, USA) and stored at −20 °C prior to further analysis. The quantity and quality of extracted DNA were measured using a NanoDrop NC2000 spectrophotometer (Thermo Fisher Scientific, Waltham, MA, USA) and agarose gel electrophoresis, respectively. PCR amplification of the V5–V7 region of the bacterial 16S rRNA gene was performed using the forward primer 338F (5′-AACMGGATTAGATACCCKG-3′) and the reverse primer 806R (5′-ACGTCATCCCCACCTTCC-3′). PCR amplicons were purified with Vazyme VAHTSTM DNA Clean Beads (Vazyme, Nanjing, China) and quantified using the Quant-iT PicoGreen dsDNA Assay Kit (Invitrogen, Carlsbad, CA, USA). After the individual quantification step, amplicons were pooled in equal amounts using the Illlumina NovaSeq platform at Shanghai Personal Biotechnology Co., Ltd. (Shanghai, China). Bioinformatic analyses including primer removal, quality filtering, denoising, sequence assembly, and chimera removal were performed using the analytical software Cutadapt (v2.3), Vsearch (v2.13.4_linux_x86_64), and QIIME2 (2019.4). The assembled and filtered sequences obtained from the above steps were aligned against a reference database to generate the final valid datasets.

### 4.8. Data Analysis

SPSS 22.0 software (IBM, Armonk, NY, USA) was used to compare differences among data. The paired-samples *t*-test was applied for pairwise comparisons (*p* value ≤ 0.05), while one-way analysis of variance (ANOVA) with LSD tests was used for comparisons involving three or more groups (*p* value ≤ 0.05). Bar charts were generated using GraphPad Prism 8 (GraphPad Software, Boston, MA, USA). The soil microbial co-occurrence network was constructed based on the OTU abundance data of soil microorganisms. Pearson’s correlation analysis (OmicStudio tools at https://www.omicstudio.cn/tool/62, accessed on 2 November 2025.) was performed to calculate the relationships among data [78], with a threshold of │Pearson’s correlation│ ≥ 0.9 and *p* value ≤ 0.05 for data screening [79,80]. The correlations between microorganisms and leaf nutrient contents were analyzed using Spearman’s correlation analysis (OmicStudio tools at https://www.omicstudio.cn/tool/62, accessed on 22 January 2026), with *p* value < 0.05 as the screening criterion (R version 4.0.3, ggplot2: 3.3.5, OmicStudioClassic: 1.0.6) [81]. Finally, Gephi 0.9.2 and Adobe Photoshop (version 21.0.1, Adobe Systems, Mountain View, CA, USA) were used to produce the figures.

## 5. Conclusions

Against the backdrop of intensifying global environmental and economic change, the rising prevalence of alien biological invasions has become an unavoidable challenge for countries and regions worldwide. A better understanding of how nitrogen deposition influences interactions between alien invasive plants and native plants is critical for predicting plant invasion dynamics and implementing effective management strategies. The results of this study demonstrate that, under exogenous nitrogen addition, *S. rostratum* gained a significant competitive advantage over *S. nigrum*: Its superior carbon assimilation and spatial resource acquisition capabilities secured its dominant position in interactions with the native species; its “low-acquisition, high-consumption” nutrient strategy endowed it with a strong ability to rapidly adapt to environmental change; and its stable and diverse rhizosphere microbial community likely promoted nutrient cycling in rhizosphere soil and enhanced root nutrient uptake.

## Data Availability

The original contributions presented in the study are included in the article/Supplementary Materials. Further inquiries can be directed to the corresponding authors.

## Supplementary Materials

The following supporting information can be downloaded at: https://www.mdpi.com/article/10.3390/plants15050687/s1, Table S1: Network attributes; Table S2: Genus and Phylum.
